# Incidence and Genetic Characterization of *Gongylonema pulchrum* in Cattle Slaughtered in Mazandaran Province, Northern Iran

**Published:** 2010-06

**Authors:** A Halajian, A Eslami, N Salehi, J Ashrafi-Helan, H Sato

**Affiliations:** 1 Department of Parasitology, Faculty of Veterinary Medicine, Tehran Science and Research Branch, Islamic Azad University, Tehran, Iran; 2 Department of Parasitology, Faculty of Veterinary Medicine, University of Tabriz, Tabriz, Iran; 3 Laboratory of Veterinary Parasitology, Faculty of Agriculture, Yamaguchi University, 1677-1 Yoshida, Yamaguchi 753-8515, Japan

**Keywords:** *Gongylonema pulchrum*, Cattle, Iran, rDNA, Diagnosis

## Abstract

**Background:**

The gullet worm, *Gongylonema pulchrum* Molin, 1857, is a thread-like spirurid nematode found in a variety of mammals worldwide. Its incidences in Iranian cattle of different breed or age have not been reported. The aims of the present study are to disclose the infection status of *G. pulchrum* in cattle slaughtered in northern region of Iran.

**Methods:**

Full-length esophagi of cattle of 97 native dairy breed and 41 Holstein-Friesian breed were collected at four local abattoirs in Mazandaran Province, northern Iran, from March 2006 to August 2007, and were examined parasitologically. Eight overlapping segments of the small- and large-subunits of rDNA were amplified by PCR, and the obtained nucleotide sequences were characterized.

**Results:**

The incidences of *G. pulchrum* in female and male native dairy breed were 38.9% and 24.0%, respectively, whereas those in female and male Holstein-Friesian breed were 4.2% and 0%, respectively. The first internal transcribed spacer (ITS1) region of *G. pulchrum* rDNA showed an intra-individual variation in the sequence and length, and the variation was ascribed to some unstable repeats of "A" or "CA".

**Conclusion:**

Distinct incidences of *G. pulchrum* infection in native dairy breed and Holstein-Friesian breed might be ascribed to different animal husbandry manners for each breed in Iran; the former breed grazes freely in the pasture, but the latter breed is usually held in a pen. The rDNA sequence of Iranian *G. pulchrum,* obtained for the first time by us, might facilitate a reliable species identification of the parasite with a wide spectrum of morphological variations.

## Introduction

The gullet worm, *Gongylonema pulchrum* Molin, 1857, is a thread-like spirurid nematode found worldwide and in a variety of mammals such as sheep, goats, cattle, zebus, buffalo, camels, donkeys, cervids, pigs, equids, bears, rodents, and primates (1, 2). In Iran, this gullet worm has been reported in cattle (3, 4), sheep (5), buffalo, goats, wild boars (6), donkeys (7), and human beings (8). Anwar et al. (4) recorded *G. pulchrum* infection in 49.7% of 555 cattle slaughtered at the central Tehran abattoir in the 1970s. Eslami and Farsad-Hamdi (6) found *G. pulchrum* infection in 35% of 57 wild boars collected in the northern, northeastern, and southwestern parts of Iran.

The aims of the present study are to disclose the infection status of *G. pulchrum* in cattle slaughtered in northern region of Iran, with special reference to incidences of *G. pulchrum* in cattle of different breed and age, and to characterize genetically the parasite collected in Iran for the first time.

## Materials and Methods

Full-length esophagi of 138 cattle (97 native dairy breed and 41 Holstein-Friesian breed) were collected at four abattoirs in Mazandaran Province, northern Iran, from March 2006 to August 2007. These abattoirs were located in the Ramsar, Tonekabon, Chaloos, and Noshahr regions facing the Caspian Sea. Data on breed, sex, age, clinical condition, animal husbandry manner, and date of slaughter were recorded for each animal.

The collected esophagi were brought to the Department of Parasitology, Faculty of Veterinary Medicine, Tehran Science and Research Branch, Islamic Azad University, Tehran, Iran, and cut open longitudinally. After peeling the mucosal layers from the underlying tissues, the mucosal surface was carefully checked with the naked eye. The full length of each esophagus was divided into three equal parts (anterior, middle, and posterior), and the number of worms found in each part was recorded. Individual worms were carefully removed from the esophageal epithelium using fine forceps. The collected worms were relaxed in tap water, then fixed in 70% ethanol-5% glycerin solution. The esophageal tissue from some cases were fixed in 10% neutral-buffered formalin, and 5-µm thick paraffin sections were cut and stained with hematoxylin-eosin according to the standard technique.

Six female and six male parasites fixed in alcohol-gylcerin solution were observed under a light microscope, and figures were drawn with the aid of a *camera lucida*. Measurements were performed on these drawn figures, using a digital curvimeter type S (Uchida Yoko, Tokyo, Japan) when necessary. The parasites used for morph-ological examination were deposited in the National Science Museum, Tokyo, Japan (Nos. NSNT-As 3501-3505).

**Fig. 1:**
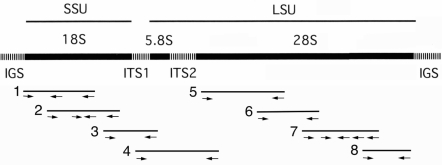
Schematic strategy to amplify and sequence the rDNA of *Gongylonema pulchrum*. Eight overlapping segments (shown here as numbered bar) were amplified using 8 primer combinations (Table 1), and sequenced by these primers for amplifying and additional primers for sequencing (see text). Small arrows show primers for sequencing. Any length shown here is not proportional to the substantial length of each rDNA region.

Following repeated washings in pure water, parasite DNA was extracted separately from two-fixed female *G. pulchrum* using an Illustra™ tissue & cells genomicPrep Mini Spin Kit (GE Healthcare UK, Buckinghamshire, UK) according to the instructions of the manufacturer. PCR amplification of overlapping rDNA fragments was performed (Fig. 1), using different primer combinations (Table 1) as described previously (9). PCR products for sequencing were purified using a High Pure PCR Cleanup Micro Kit (Roche Diagnostics GmbH, Mannheim, Germany). In addition to primers for amplifying, following primers were used for sequencing: segment 1, SSU9R (5'-AGCTGGAATTACCGCGGCTG-3'); segment 2, SSU24F (5'-AGAGGTGAAATTCGTGGACC-3' and 18S-1192R/20 (Table 1); and for segment 7, NLF1999/19 (5'-CCGCAKCAGGTCTCCAAG-3'), 28S-2132R/20 (5'AGAGGCTGTTCACCTTGGAG-3'), and NLR2362/20 (5'-ACATTCAGAGCACTGGGCAG-3'). After direct sequencing of PCR amplicons <bibr>9?, sequences were assembled manually with the aid of the CLUSTAL W multiple alignment program (10). For an rDNA segment containing the first and second internal transcribed spacer (ITS1 and ITS2) regions, the amplicon was cloned into a plasmid vector, pTA2 (Targe Clone™; TOYOBO, Osaka, Japan), and transformed into *Escerichia coli* JM109 (TOYOBO) according to the instructions of the manufacturer. Following propagation, plasmid DNA was extracted using a NucleoSpin® Plasmid kit (MACHEREY-NAGEL GmbH, Düren, Germany), and inserts from multiple independent clones were sequenced using universal M13 forward and reverse primers

**Table 1:** Primers used to amplify 9 overlapping segments of rDNA of *G. pulchrum*

Segment			Length ofamplicon (bp)

No.	Forward	Reverse	
1	F-47(5'- CCCGATTGATTCTGTCGGC-3')SSU22F	18S-1192R/20 (5'-CAGGTGAGTTTTCCCGTGTT-3')	1,211
2	(5'-TCCAAGGAAGGCAGCAGGC-3')SSU23F	NSR1787/18 (5'-CGACGGGCGGTGTGTACA-3')	1,230
3	(5'-ATTCCGATAACGAGCGAGACT-3')NC5(ITS1)/F	NC13(ITS1)/R (5'-GCTGCGTTCTTCATCGAT-3')	918–929
4	(5'- AGGTGAACCTGCGGAAGGATCATT-3')28S/F	28S-408R/20 (5'-TTCACGCCCTCTTGAACTCT-3')	1,202–1,213
5	(5'-AGCGGAGGAAAAGAAACTAA-3')28S-839F/20	28S-1270R/22 (5'-CAGCTATCCTGAGGGAAACTTC-3')	1,191
6	(5'-TATCCGACCCGTCTTGAAAC-3')28S-1449F/27	28S-1670R/20 (5'-TACCACCAAGATCTGCACCA-3')	955
7	(5'-GAAGTCGGAATCCGCTAAGGAGTGTG-3')NLF2551/21	NLR2781/19 (5'-CCGCCCCAGYCAAACTCCC-3')	1,537
8	(5'-GGGAAAGAAGACCCTGTTGAG-3')	NLR3284/21 (5'-TTCTGACTTAGAGGCGTTCAG-3')	749

The relationships between the infection rate and several parameters related to the examined cattle were assessed by the contingency table method and regression analysis using commercially available statistical software (StatView® ver.5; SAS Institute Inc., North Carolina, USA). A *P* value less than 0.05 denoted statistical significance.

## Results

The recovery of *G. pulchrum* from 138 cattle (97 native dairy breed and 41 Holstein-Friesian breed) is shown in (Table 2). Only a single female Holstein-Friesian breed was infected with *G. pulchrum*, whereas 38.9% (28 of 72) female and 24.0% (6 of 25) male native dairy cattle were infected with the gullet worm. The incidence of the gullet worm in native dairy breed, particularly female cattle, was positively correlated with the host age (P<0.05). The gullet worms were collected in 25.9% (7 of 27) of cattle in winter, 58.3% (7 of 12) of cattle in spring, 33.3% (5 of 15) of cattle in summer, and 34.9% (15 of 43) of cattle in autumn. The incidences of the gullet worms in native dairy breed were not different by sex and season. Worm recovery ranged from 1 to 24, and the geomean was 2.3 in female and 1.6 in male native dairy cattle. In total, 26 worms were collected from the anterior, 41 worms from the middle, and 47 worms from the posterior part of the esophagus of native dairy cattle. All 4 worms were collected from the posterior part of the esophagus of the single *G. pulchrum*-infected Holstein-Friesian breed.

**Table 2:** Recovery of *Gongylonema pulchrum*from the esophagus of cattle in Iran*

	Approximate age of animals (years)					
	1	2	3	4	≥5	Total
Holstein-Friesian breed						
Female						
Number of animals examined	3	7	6	6	2	24
Number of infected animals	0	0	0	1	0	1
Incidence (%)	0	0	0	16.7	0	4.2
Intensity	0	0	0	4	0	4
Male						
Number of animals examined	0	2	13	2	0	17
Number of infected animals	0	0	0	0	0	0
Incidence (%)	0	0	0	0	0	0
Intensity	0	0	0	0	0	0
Breed total						
Number of animals examined	3	9	19	8	2	41
Number of infected animals	0	0	0	1	0	1
Incidence (%)	0	0	0	12.5	0	2.4
Native dairy breed						
Female						
Number of animals examined	5	12	14	26	15	72
Number of infected animals	0	2	5	8	13	28
Incidence (%)	0	16.7	35.7	30.8	86.7	38.9
Intensity	0	3 & 24	1.1 (1–2)	2.2 (1–11)	2.5 (1–9)	2.3 (1–24)
Male						
Number of animals examined	4	3	11	5	2	25
Number of infected animals	2	1	2	1	0	6
Incidence (%)	50.0	33.3	18.2	20.0	0	24.0
Intensity	1 & 4	1	1 & 2	2	0	1.6 (1–4)
Breed total						
Number of animals examined	9	15	25	31	17	97
Number of infected animals	2	3	7	9	13	34
Incidence (%)	22.2	20.0	28.0	29.0	76.5	35.1

* Intensity is expressed as mean of worm recovery (range, when available).

**Fig. 2:**
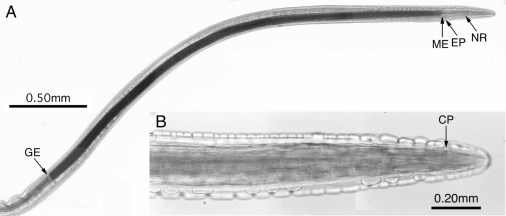
Anterior end of female *Gongylonema pulchrum* from a cattle in Iran. (A) Low power view of the anterior end showing a whole length of esophagus. EP, Excretory pore; GE, end of grandular esophagus; ME, end of muscular esophagus; and NR, nerve ring. (B) High power view of the anterior end showing cervical lateral papilla (CP) and prominent cuticular plaques irregularly arranged in longitudinal rows.

Morphological examination of the collected worms showed characteristic cephalic and caudal morphologies of *G. pulchrum* (Fig. 2), and the measurements of 6 worms of each sex are shown in Table 3. Histological examination detected mild local inflammation around the worm trail, but not directly around the worms, in the epithelial layers and the lamina propria, along with local acantholysis and hyperkeratosis.

**Table 3:** Comparison of measurements of *Gongylonema pulchrum* collected from cattle (in mm)

Sex of worms	Male		
Locality	Iran		Northern part of Japan
Reference	The present study	Baylis, 1925 (11)	Kudo et al., 1992 (12)
Number of worms examined	6	?	60
**Body length**	36.7–48.6 (41.9±4.8)	12–62	24.1–52.4
**Max. body width**	0.22–0.26 (0.24±0.02)	0.14–0.36	0.208–0.296
**Pharynx**	0.045–0.056 (0.048±0.005)	0.040–0.075	0.042–0.066
**Length of esophagus**	4.96–6.08 (5.58±0.43)	3.0–7.0	4.89–7.44
** Muscular portion**	0.55–0.65 (0.61±0.04)	0.40–0.78	0.478–0.697
** Glandular portion**	4.40–5.47 (4.97±0.43)	—	4.35–6.78
Cervical lateral papillae*	0.134–0.171 (0.150±0.013)	0.075–0.19	0.116–0.186
Nerve ring*	0.266–0.322 (0.298±0.020)	0.29–0.35	0.264–0.352
Excretory pore*	0.495–0.599 (0.537±0.039)	0.30–0.65	0.408–0.616
**Left spicule**	10.60–27.86 (18.87±6.46)	4.0–23.0	11.1–22.7
**Right spicule**	0.137–0.168 (0.157±0.014)	0.084–0.18	0.118–0.160
**Gubernaculum**	0.109–0.140 (0.130±0.018)	0.07–0.12	0.106–0.142
**Number of precloacal papillae**	5—6	—	5—7
**Number of postcloacal papillae**	5—6	—	5—6
**Tail length**	0.172–0.336 (0.275±0.062)	0.22–0.35	0.240–0.376
			


**Sex of worms**	**Female**		
**Locality**	Iran		Northern part of Japan
**Reference**	The present study	Baylis, 1925 (11)	Kudo et al., 1992 (12)
**Number of worms examined**	6	?	60

**Body length**	68.5–107.3 (82.4±14.4)	37–145	46.0–111.5
**Max. body width**	0.29–0.38 (0.32±0.04)	0.19–0.53	0.272–0.424
**Pharynx**	0.045–0.064 (0.055±0.007)	0.040–0.075	0.044–0.073
**Length of esophagus**	6.46–8.39 (7.58±0.72)	6.0–9.0	5.80–9.46
** Muscular portion**	0.63–0.88 (0.80±0.10)	0.48–0.95	0.576–0.936
** Glandular portion**	5.83–7.52 (6.77±0.63)	—	5.20–8.60
Cervical lateral papillae*	0.168–0.224 (0.189±0.020)	0.13–0.22	
Nerve ring*	0.324–0.453 (0.382±0.049)	0.25–0.40	
Excretory pore*	0.632–0.862 (0.743±0.087)	0.46–0.90	
Vulva**	2.69–5.18 (3.63±0.96)	1.95–7.0	1.97–6.06
**Tail length**	0.246–0.414 (0.307±0.058)	0.19–0.38	0.202–0.380
**Egg**	0.058–0.060 (0.059±0.001)×0.032–0.034 (0.033±0.001)	0.050–0.070×0.025–0.037	0.050–0.063×0.032–0.038

* From the anterior end.

** From the posterior end.

Except for approximately 20 nucleotides corresponding to the 5'-end of SSU rDNA, the rest of its sequence (1,782 bp in length), ITS1, 5.8S rDNA, ITS2, and more than 3,500-bp long 28S rDNA sequences were determined in this study (DDBJ/EMBL/GenBank accession no. **AB495389**). The ITS1 showed an intra-individual variation in the sequence and length (DDBJ/EMBL/GenBank accession nos. **AB495389-AB495393**). The variations in length of ITS1, from 378-bp to 389-bp, was ascribed to some unstable repeats of "A" (11 to 14 times) or "CA" (3 to 8 times). Except for the ITS1 region, the rDNA sequence of *G. pulchrum* in cattle in Iran was identical with those of the parasite in cattle in Japan (DDBJ/EMBL/GenBank accession nos. **AB513707 – AB513723**).

## Discussion

Morphological characters of the collected worms by us are identical to *G. pulchrum* described in previous reports (11–13). Distributions of *G. pulchrum* with high incidences have been fairly well documented in a variety of domestic and wild mammals in Iran (4, 6, 7). In addition, one human case has been reported in this country (8). As previously suggested by these reports, the present study has also confirmed a high prevalence of *G. pulchrum* infection in Iran. As shown in a report from Japan, the ages of examined cattle apparently affect the incidence of *G. pulchrum* infection. Kudo et al. (12) reported the incidence at 3.3% (8 of 242) in cattle 2 years old or younger, 17.0% (40 of 235) in cattle 3 years old, and 60.0% (57 of 94) in cattle 4 years old or older. In this study, accumulation of *G. pulchrum* parasitism by age was evident particularly in female native dairy cattle in Iran, which graze freely in the pasture. In contrast, pen-held Holstein-Friesian breed might have less opportunity to be infected with *G. pulchrum*.

Due to the accidental occurrence of *G. pulchrum* infection in humans, when frequently only a fragment of the parasite is recovered for the causative species identification (8, 14–16), and a stunted development of the parasite in non-bovine hosts (17–20), or a wide spectrum of its morphological variations by hosts exists (21), the molecular-based diagnosis of the species might be convenient and accurate. The rDNA of *G. pulchrum* or any other members of the family Gongylonematidae, however, have not been sequenced to date. We have presented, for the first time, approximately 6,100-bp long rDNA sequences of *G. pulchrum* in the present study (DDBJ/EMBL/GenBank accession no. **AB495389**-**AB495393**). Furthermore, we noticed no geographical variations in the rDNA sequences except for the ITS1 region between *G. pulchrum* isolated from cattle in Iran and Japan. This rDNA-based diagnosis of the species may facilitate the reliable identification of *G. pulchrum* even under limitation of sampling for morphological species diagnosis. Further, the complicated taxonomy of members of the genus *Gongylonema* (1, 2) would be solved after accumulation of rDNA datasets of members of the genus.

We believe, for a long time, that *G. pulchrum* takes a wide spectrum of mammals as natural hosts, and domestic and wild mammals share the transmission cycle by an accidental ingestion of the intermediate host, dung beetles. Our study in progress attempts to disclose the transmission dynamics or epidemiology of *G. pulchrum* between domestic and wild mammals using mitochondrial DNA markers, which is relevant to detect inraspecific polymorphism (22), and find a possibility that diverse genotypes of *G. pulchrum* may be prevalent in different mammalian groups.
